# Vaccines and the 2024 US Presidential Election

**DOI:** 10.1001/jamahealthforum.2025.5361

**Published:** 2025-12-05

**Authors:** Joshua M. Sharfstein, Erik Westlund, Shannon Frattaroli, Adam S. Levine, Keshia Pollack Porter

**Affiliations:** 1 Department of Health Policy and Management, Johns Hopkins Bloomberg School of Public Health, Baltimore, Maryland; 2 Department of Biostatistics, Johns Hopkins Bloomberg School of Public Health, Baltimore, Maryland; 3 SNF Agora Institute, Johns Hopkins University, Baltimore, Maryland

## Abstract

This survey study reports on the vaccine-related views of voters surveyed soon after the 2024 US presidential election.

## Introduction

The second Trump administration is transforming US vaccine policy by altering public communications,^1^ vaccine advisory committees,^2^ processes for vaccine approval,^3^ and vaccine recommendations.^4^ To provide insight on public alignment with these changes, we report on the vaccine-related views of voters surveyed soon after the 2024 US presidential election.

## Methods

We added 3 questions to the AmeriSpeak Omnibus survey, administered from November 21 to 25, 2024, among a nationally representative sample of US adults. One question asked respondents to choose up to 3 issues from a list of 12 that mattered for their 2024 vote. Other questions provided a 5-point Likert scale to assess support for policy goals, which included “continued government support for safe and effective vaccines” and “removing school vaccination requirements for children.” We analyzed results among voters in the 2024 presidential election by candidate choice, age, region of the country, education, income, and race and ethnicity. Additional details are in the eMethods in Supplement 1.

The study was approved by the institutional review board at the Johns Hopkins Bloomberg School of Public Health. The survey key indicators correspond to the guidelines of the American Association for Public Opinion Research Transparency Initiative (eMethods in Supplement 1).

## Results

In total, 1236 individuals at least 18 years old from across the US responded to the questions, of whom 964 voted in the 2024 election. Very few voters (3.0%) selected vaccines as a major factor in their 2024 presidential vote (1.5% of Donald Trump voters and 4.4% of Kamala Harris voters) (Table 1). Most voters (76.3%) backed government support for safe and effective vaccines (58.7% of Trump voters and 90.3% of Harris voters). A minority of voters (18.5%) supported removing vaccination requirements for school (32.5% of Trump voters and 7.3% of Harris voters). Rates of agreement or disagreement with vaccine policies differed by education levels (Table 2).

**Table 1.** Issues That Mattered and Policy Support by Voter Group in the 2024 Presidential Election

Survey question	% (SE)		
	All	Trump voters	Harris voters
**Which issues, if any, mattered to you during the 2024 presidential election? Choose up to 3.**			
Inflation	47.3 (2.1)	70.9 (3.0)	28.3 (2.6)
Immigration	38.9 (2.1)	70.4 (3.0)	13.7 (1.8)
Democracy	34.8 (1.9)	11.3 (2.1)	54.5 (2.8)
Abortion	33.8 (1.9)	17.3 (2.4)	47.9 (2.8)
Health care	27.1 (1.8)	11.9 (1.8)	38.9 (2.7)
Crime	22.8 (1.8)	39.6 (3.3)	9.9 (1.5)
Foreign policy	16.7 (1.5)	20.5 (2.6)	12.5 (1.7)
Education	14.9 (1.5)	7.6 (1.8)	21.1 (2.4)
Inequalities and inequities	14.4 (1.3)	0.9 (0.4)	25.0 (2.3)
Jobs	12.9 (1.5)	18.0 (2.6)	9.0 (1.7)
Poverty	11.0 (1.3)	7.9 (1.7)	13.3 (2.0)
Vaccines	3.0 (0.7)	1.5 (0.6)	4.4 (1.2)
Total No. unweighted	964	NA	NA
Total No. weighted	943	NA	NA
**Here is a list of specific policy goals. To what extent would you support or oppose them?**			
Continue government support for safe and effective vaccines			
Strongly support	44.2 (2.1)	21.9 (2.7)	62.3 (2.8)
Support	32.1 (2.0)	36.8 (3.2)	28.0 (2.6)
Neither support nor oppose	17.4 (1.7)	30.2 (3.2)	7.2 (1.5)
Oppose	2.5 (0.6)	5.3 (1.3)	0.2 (0.2)
Strongly oppose	2.6 (0.7)	5.2 (1.6)	0.4 (0.3)
Total No. unweighted	955	NA	NA
Total No. weighted	932	NA	NA
Removing school vaccination requirements for children			
Strongly support	9.4 (1.6)	16.0 (3.0)	4.3 (1.5)
Support	9.1 (1.3)	16.5 (2.6)	3.0 (1.1)
Neither support nor oppose	16.9 (1.6)	28.3 (3.0)	7.6 (1.4)
Oppose	23.9 (1.7)	25.4 (2.6)	22.5 (2.2)
Strongly oppose	39.7 (2.0)	13.7 (2.0)	60.7 (2.8)
Total No. unweighted	958	NA	NA
Total No. weighted	934	NA	NA

Abbreviation: NA, not applicable.

**Table 2.** Support for Vaccine-Related Policies by Educational Attainment Among Voters in the 2024 Presidential Election

Survey question	% (SE)				
	Less than high school	High school graduate or equivalent	Some college/associate’s degree	Bachelor’s degree	Postgraduate study/professional degree
**Which issues, if any, mattered to you during the 2024 presidential election? Choose up to 3. (964 unweighted; 943 weighted)**					
Vaccines	6.3 (3.1)	2.3 (1.0)	1.8 (0.8)	3.1 (1.2)	4.5 (1.6)
**Continue government support for safe and effective vaccines (955 unweighted; 932 weighted)**					
Strongly support	35.2 (6.0)	34.9 (3.1)	44.1 (3.1)	45.2 (3.4)	59.2 (3.8)
Support	29.6 (5.7)	33.1 (3.1)	30.2 (2.9)	38.9 (3.3)	25.6 (3.3)
Neither support nor oppose	23.3 (5.3)	27.8 (2.9)	16.5 (2.3)	11.3 (2.1)	10.1 (2.3)
Oppose	0.0	1.5 (0.8)	4.4 (1.3)	1.8 (0.9)	2.7 (1.2)
Strongly oppose	6.2 (3.0)	2.5 (1.0)	3.7 (1.2)	1.7 (0.9)	0.8 (0.7)
**Removing school vaccination requirements for children (958 unweighted; 934 weighted)**					
Strongly support	37.0 (6.1)	13.7 (2.3)	5.4 (1.4)	5.9 (1.6)	3.8 (1.5)
Support	12.1 (4.1)	12.5 (2.2)	9.8 (1.9)	5.4 (1.5)	6.9 (1.9)
Neither support nor oppose	23.5 (5.3)	18.5 (2.6)	22.2 (2.6)	14.6 (2.4)	7.6 (2.0)
Oppose	9.9 (3.7)	22.4 (2.7)	26.9 (2.8)	27.7 (3.0)	22.0 (3.2)
Strongly oppose	11.8 (4.0)	32.9 (3.1)	35.7 (3.0)	45.1 (3.4)	58.2 (3.8)

## Discussion

In this survey study, very few US voters considered vaccines an important issue in the 2024 president election, but voters generally supported the government’s role in ensuring safe and effective vaccines and requiring children to be vaccinated for school. The partisan divide on vaccines reflects solid support among Trump voters compared to high support among Harris voters. A March 2025 poll showed that 68% of Republicans and 90% of Democrats support school vaccination requirements^5^—similar to the present results. Opposition was explained by support for parental choice in deciding whether to vaccinate their children, concerns over safety, and other factors. In this study, more than one-third of Trump voters did not favor a government role in facilitating safe and effective vaccines, with voters with only high school education disproportionately represented in this group.

This survey, conducted before the new administration took office, could not assess public opinion on specific changes to vaccine policy. But the results suggest that most individuals in the US may not support the major shifts that have taken place; consistent with this hypothesis, a recent national survey found that just 24% of those polled believe the administration’s actions on vaccines are supported by science.^6^ An outstanding question is whether voters will increase their focus on vaccine policy or continue to prioritize other issues. The answer may determine the fate of the current trajectory of policy changes.

## Notes

Supplement 1.
eMethods

eReferences

Supplement 2.
Data Sharing Statement
