# Supplementary material for: Vaccines and the 2024 US Presidential Election
Source: JAMA Health Forum. 2025 Dec 5;6(12):e255361. doi: 10.1001/jamahealthforum.2025.5361 (PMC12681029; doi:10.1001/jamahealthforum.2025.5361)
Supplement: Supplement 1. — eMethods eReferences [file jamahealthforum-e255361-s001.pdf]

Supplemental Online Content

Sharfstein JM, Westlund E, Frattaroli S, Levine AS, Pollack Porter K. Vaccines and the 2024 US presidential election. *JAMA Health Forum*. 2025;6(12):e255361. doi:10.1001/jamahealthforum.2025.5361

eMethods

eReferences

This supplemental material has been provided by the authors to give readers additional information about their work.

© 2025 Sharfstein JM et al. *JAMA Health Forum*.

eMethods

Survey Administration

This study was conducted using the NORC AmeriSpeak Omnibus online panel. Three custom questions were included in the November 2024 wave. Responses were collected from 1,236 adults aged 18 and older between November 21 and 25, 2024.

Panel Design

The AmeriSpeak Panel is a probability-based panel developed and managed by NORC at the University of Chicago. It is designed to be representative of the U.S. household population, covering approximately 97% of U.S. households, including rural and low-income populations. Panelists are recruited through address-based sampling and in-person outreach. For more information on the AmeriSpeak Panel's sampling and recruitment methodology, see:

https://amerispeak.norc.org/us/en/amerispeak/about-amerispeak/panel-design.html

Weighting

Survey weights provided by NORC adjusted for unequal probabilities of selection and nonresponse. Post-stratification aligned the sample with the most recent Current Population Survey benchmarks across gender, age, race/ethnicity, education, and geographic region. All analyses used these weights and were conducted in R using the survey package (Lumley, 2024).

The full survey instrument and analysis code are available at:

https://github.com/JHBiostatCenter/norc-amerispeak-omnibus-survey

Analysis

Data were analyzed using the *tidyverse* R package. Weights were applied using the *survey* package for R with the sample weights provided by NORC, so estimates represented U.S. adults. A Cramers V test was utilized for test of association of the demographic factors with vaccine policy answers, and a Somers D test was utilized for factors with ordinal variables.

AAPOR Transparency Initiative

For our survey, the key indicators in the AAPOR Transparency Initiative are:

Released sample	7215
Completes	1236
Panel recruitment rate:	27.4%
Panel retention rate:	77.2%
Survey completion rate	17.1%
AAPOR3 response rate:	3.6%
Margin of error:	3.65%
Design effect:	1.72

© 2025 Sharfstein JM et al. *JAMA Health Forum*.

Statistical Tests of Association for Demographic Factors

Statistical Notes

Standard errors: Cramér's V p-values are derived from the weighted chi-square test via svychisq() in R's survey package[1]. Somers' D p-values are calculated using the SomersDelta function in the DescTools package which computes asymptotic standard errors accounting for survey weights (Signorell, 2025)[2].

eReferences

1. T. Lumley (2024) "survey: analysis of complex survey samples". R package version 4.4.
2. Signorell A (2025). DescTools: Tools for Descriptive Statistics. R package version 0.99.60.

© 2025 Sharfstein JM et al. *JAMA Health Forum*.
