# Supplementary material for: Vaccines and the 2024 US Presidential Election
Source: JAMA Health Forum. 2025 Dec 5;6(12):e255361. doi: 10.1001/jamahealthforum.2025.5361 (PMC12681029; doi:10.1001/jamahealthforum.2025.5361)
Supplement: Supplement 2. — Data Sharing Statement [file jamahealthforum-e255361-s002.pdf]

Data Sharing Statement

Sharfstein. Vaccines and the 2024 US Presidential Election. *JAMA Health Forum*. Published December 05, 2025. doi:10.1001/jamahealthforum.2025.5361

Data
Data available: Yes

Additional Information
Tables with demographic and political characteristics of 2024 voters by vote choice and vaccine-related responses by demographic categories among 2024 voters, as well as statistical tests of association, are available from the authors by request.
